# Editorial

**Published:** 1851-07

**Authors:** 


### THE MEDICAL EXAMINER.

PHILADELPHIA, JULY, 1851.

In the account of the late proceedings of the American Medical Association, published in our June number, the attention of our readers was perhaps directed to some remarkable circumstances connected with the presentation of the report of the Committee on Obstetrics. After this report had been read by the chairman, Dr. STORER, of Boston, the accuracy of certain statistical facts, embodied in it, was called in question by Dr. ROBERTSON, of South Carolina. These facts had been extracted from a paper by Dr. H. A. RAMSEY, of Lincoln county, Georgia, published in the last October number of this journal. Dr. Robertson moved "that the statistics, alluded to in the report, be stricken out, as the author of them was not a reliable man." And it appears that this motion, in substance, prevailed, and that "Dr. Storer erased the statistics referred to."

We confess that these proceedings struck us as somewhat extraordinary. We have no personal knowledge of either of the gentlemen named, and we write in the dark as to the under-current of feeling which may have prevailed on the occasion. But, forming our judgment from the record, we cannot but dissent from the decision which was made. Whatever the merits of the case, the ordinary forms of justice were disregarded in the condemnation of an unheard, absent person; and there appears to us to have been in every way a want of propriety in the time, the place, and the tribunal, which were selected for the trial of this question of credibility. The affair seems to have been very much a 'snap judgment.'

We have received from Dr. Ramsey a letter on the subject, in which he indignantly repels the assault upon his veracity. He has forwarded to us a number of testimonials as to his reliability and integrity, from his professional brethren and others of his neighborhood. And he also states, that he has invited Dr. Robertson to substantiate his charge before a board of professional referees, in whose decision he will entirely acquiesce.

We have felt it due to our correspondent to allow him to be heard in reply to this attack. Let us, however, add, that we take no part whatever in the personal controversy between the gentlemen in question, and that our remarks apply only to the manner in which it originated.

---

### OBITUARY.

DIED,—At Philadelphia, June 24th, DR. SPENCER SERGEANT, second son of the *Hon. John Sergeant.* Dr. Sergeant was lately one of the resident physicians at the Pennsylvania Hospital, where his quiet and faithful discharge of his duties, as well as his amiable and gentlemanly deportment, endeared him to all connected with that institution. Although young in years, he had given bright promise of eminence and usefulness. Many of our readers will remember the able reports that emanated from his pen during his official connection with the Hospital, a large proportion of which, with his judicious practical remarks upon them, were extensively copied both at home and abroad. To his many friends, as well as to the profession, his early death will cause a void not easily to be filled.

